# Exercise intervention prevents early aged hypertension-caused cardiac dysfunction through inhibition of cardiac fibrosis

**DOI:** 10.18632/aging.204077

**Published:** 2022-05-23

**Authors:** Yi Hong, Ai-Lun Yang, James K.S. Wong, Kunanya Masodsai, Shin-Da Lee, Yi-Yuan Lin

**Affiliations:** 1 The First Rehabilitation Hospital of Shanghai, Shanghai, China; 2 Department of Sports Sciences, University of Taipei, Taipei, Taiwan; 3 Department of Cardiology, Asia University Hospital, Taichung, Taiwan; 4 Department of Bioinformatics and Medical Engineering, Asia University, Taichung, Taiwan; 5 Faculty of Sports Science, Chulalongkorn University, Bangkok, Thailand; 6 Department of Physical Therapy, Graduate Institute of Rehabilitation Science, China Medical University, Taichung, Taiwan; 7 Department of Physical Therapy, Asia University, Taichung, Taiwan; 8 School of Rehabilitation Medicine, Weifang Medical University, Shandong, China; 9 Department of Exercise and Health Science, National Taipei University of Nursing and Health Sciences, Taipei, Taiwan

**Keywords:** myocardial fibrosis, hypertension, treadmill training, aging

## Abstract

Background: An inappropriate accumulation of fibrillar collagen is a common pathologic feature of early aged hypertensive heart disease, but little information regarding the effects of exercise training on cardiac fibrosis in hypertension is available. The purpose of this study was to evaluate the effects of exercise training on cardiac fibrotic pathways in early aged hypertensive rats.

Methods: Masson’s trichrome staining and Western blotting were performed on the excised left ventricle from twenty male spontaneously hypertensive rats at age of 48 weeks, which were randomly divided into either a sedentary hypertensive group (SHR) or exercise hypertensive group (SHR-EX, running on a treadmill running occurred 5 days/week for 60 min/day, for 12 weeks), and from age-matched male Wistar–Kyoto normotensive controls (WKY).

Results: Interstitial fibrosis was reduced in the SHR-Ex group when compared with the SHR group. The fibrotic-related protein levels of AT_1_R, FGF23, LOX-2, TGF-β, CTGF, p-Smad 2/3, MMP-2/TIMP-2, MMP-9/TIMP-1, uPA and collagen I were decreased in the SHR-EX group, when compared with the SHR group.

Conclusions: Exercise training suppresses early aged hypertensive heart-induced LOX-2/TGF-β-mediated fibrotic pathways associated with decreasing AT_1_R and FGF23, which might provide a new therapeutic effect for exercise training to prevent adverse cardiac fibrosis and myocardial abnormalities in early aged hypertension.

## INTRODUCTION

Advancing age is associated with the prevalence of hypertension, which is the leading cause of death among older adults [1]. Hypertension is a risk factor for heart failure, and its prevalence continues to rise worldwide [2, 3]. The normal ejection fraction and systolic dysfunction are the most common cardiac complications of hypertension, which have been associated with widely dispersed apoptosis and fibrosis, as well as, potentially, development of heart failure [4, 5].

Sustained hypertension not only induces renovascular remodeling, but also induces myocardial fibrosis by altering extracellular matrix components [5]. Moreover, hypertension-induced myocardial fibrosis is a critical pathological process in the development of heart failure [6]. In response to various extracellular stimuli, fibroblast growth factor 23 (FGF23) associates with cardiac fibrosis and inflammation, moreover, angiotensin II through its type I receptor (AT_1_R) stimulates FGF-23, while the afferent pathways potentially moderating FGF-23-related cardiotoxicity [7]. Additionally, lysyl oxidase like-2 (LOXL2) activation is essential for cardiac fibrosis and heart failure development in animal and human models [8]. LOXL2 perturbed epithelial-mesenchymal transition, extracellular matrix deposition, themselves become factors that exacerbate the heart failure state [9]. Similarly, LOXL2 acts downstream of the transforming growth factor-beta (TGF-β) signaling which exacerbates myocardial fibrosis and heart failure induced by abdominal aortic coarctation in rats [10]. TGF-β is closely related to heart failure and myocardial damage, repair and remodeling, through stimulation induces myofibroblast differentiation and favors excessive accumulation of extracellular matrix proteins [11]. Moreover, TGF-β is a potent inducer of connective tissue growth factor (CTGF) to promote fibrogenic pathway activation through Smad protein, which has been shown to promote an extracellular matrix production and proliferation in connective tissues, leading to the progression of cardiac interstitial fibrosis [12, 13]. Dysregulation of Matrix metalloproteinases (MMPs) is involved in myocardial extracellular matrix remodeling and cardiac collagen deposition. The MMP-9 and MMP-2 deficiency expression can inhibit prevented myocardial collagen accumulation, and attenuates left ventricular enlargement, left ventricular dilatation and dysfunction after acute MI [14–16]. Within the extracellular matrix, the TIMP1 and TIMP2 are endogenous specific inhibitors of MMP-2 and MMP-9 against cardiovascular diseases [17]. Moreover, inhibition of Urokinase plasminogen activator (uPA) is showed to reduces left ventricular remodeling in uPA−/− mice [18]. Previous studies showed that excessive extracellular matrix resulting from an imbalance between synthesis and degradation of collagen. Indeed, several studies have evidenced enhanced expression and activity of cardiac LOX-2, TGF-β and FGF 23 and their relationship with adverse myocardial remodeling and dysfunction [9, 19, 20]. Hence, we hypothesized that early aged hypertensive-induced LOX-2/TGF-β-mediated myocardial fibrosis by AT_1_R and FGF 23 signaling in this process.

Physical activity is a critical component of lifestyle therapy for the primary prevention of hypertension [21, 22]. Exercise training decreased the risk for hypertension and improved cardiac function [23, 24]. Indeed, several studies have reported that exercise training alleviates cardiac fibrosis through downregulation of TGF-β and FGF23 signaling is beneficial to cardiac function [19, 20]. However, the effect of exercise training on cardiac fibrosis in early aged hypertension is not understood. Therefore, this study hypothesized that exercise training may prevent cardiac LOX-2/TGF-β-mediated fibrotic pathways associated with decreasing AT_1_R and FGF 23.

## MATERIALS AND METHODS

### Animal model

Forty-eight-week-old male Wistar Kyoto rats and twenty-eight male spontaneously hypertension rats were housed under standard laboratory conditions with a 12-h/12-h light/dark cycle and were maintained on a standard laboratory chow and had access to water *ad libitum*. This research was approved by the Institutional Animal Care and Use Committee of the University of Taipei, Taiwan (Ethical approval code: UT104005), principles specified by laboratory animal care (NIH publication).

### Exercise training

The exercise training protocol on a motor-driven treadmill (Treadmill Exerciser T408E, Diagnostic and Research Instruments Co., Taoyuan, Taiwan) were implemented according to the study of Chen [25]. Rats from the SHR-EX group had run at the speed of 15 m/min for 20 minutes day 1, and then 5 days per week based on a scale which increased by 10 minutes each day until it reached a running period of 60 minutes per session prior to the start of exercise training. As for exercise duration, the rats run at a speed of 18 m/min then gradually increased 3 m/min and maintain a pace at 27 m/min, 5 sessions per week, for 12 total weeks. In contrast, rats from the sedentary groups were placed on the stationary treadmill for the same environmental stimulation.

### Measurement of resting heart rate and blood pressure

The systolic/diastolic/mean arterial blood pressure (SBP/DBP/MAP), and heart rate of the rats was measured via a noninvasive tail-cuff blood pressure system (BP98A, Softron Co., Ltd., Tokyo, Japan). Five consecutive blood pressure readings are obtained.

### Hematoxylin-eosin staining

The heart tissues were fixed with 10% formalin solution and enclosed in paraffin. Sections of 2 μm thickness were obtained at the level of the heart, deparaffinized by hydrated, boiled using Trilogy solution (Cell Marque, Rocklin, CA, USA). The heart sections were stained with hematoxylin (Merck, Darmstadt, Germany) and treated with eosin (Merck). Following dehydration in a graded alcohol and soaked in xylene twice. Photomicrographs of heart sections were captured with an optical microscope (BX43 Olympus, Tokyo, Japan).

### Masson’s trichrome staining

For visualization of collagen fibers was performed using the Masson’s trichrome kit (Scytek Laboratories, Logan, UT, USA). In brief, the slices were soaked in an immersed Bouin’s solution and then stained in Weigert’s hematoxylin. Later the slides were immersed with acid fuchsine, phosphomolybdic acid solution and, methyl blue solution, respectively. Later the heart sections were treated with 1% acetic acid solution and dried and mounted on glass slides. Photomicrographs of heart sections were counted in least 6 separate fields × two slides × three left ventricle regions per condition (6 rat hearts in each group) were taken for data quantification using an optical microscope (BX43 Olympus, Tokyo, Japan). The digital quantification of fibrotic areas (stained blue) and myocardial areas (stained red) was performed using manual inspection (Image J, National Institute of Health, Bethesda, MD, USA).

### Protein extraction and western blot

The Bradford method (Bio-Rad Lab., Hercules, CA, USA) was used to measure protein concentration. Protein samples (40 μg/lane) were resolved by SDS polyacrylamide gel electrophoresis (SDS-PAGE) and transferred onto polyvinylidene difluoride (PVDF) membrane (Millipore, Bedford, MA, USA, 0.45 μm pore size). Block the PVDF membranes for overnight at 4°C with blocking buffer (BlockPRO, Visual Protein Biotechnology, Taipei, Taiwan) and then incubated with the primary antibodies including AT_1_R (1:1000, Novus Biologicals, Littleton, CO), FGF23, LOX-2, TGF-β, CTGF, p-Smad 2/3, MMP-2, MMP-9, TIMP-1, TIMP-2, uPA, collagen I and β-actin (1:1000, Cell Signaling Technology, Danvers, MA, USA) at overnight 4°C. Next, the PVDF membranes were treated in the second antibody solution (Jackson ImmunoResearch Laboratories, West Grove, PA, USA) and diluted 5000-fold in a TBS buffer. The protein bands were detected by ECL western Blotting luminal Reagent (Millipore Corporation, Billerica, MA, USA) and the intensities of bands were quantified using a bioimaging analyzer (LAS-3000; Fujifilm Corporation, Tokyo, Japan). The β-actin was used as the internal reference.

### Statistical analysis

All data were expressed as mean ± SD values. The statistical significance among three groups by one-way ANOVA with Tukey multiple comparison test. SPSS 25.0 software was used for analyzing and *p* < 0.05 was regarded as significant.

## RESULTS

### Cardiac characteristics

The body weight in the sedentary hypertensive group (SHR) and exercise hypertensive group (SHR-EX) were not different form the normotensive group (WKY). The WHW, LVW, WHW/BW, LVW/BW, WHW/TL and LVW/TL were increased in the SHR groups when compared with the WKY group (Table 1). The WHW, LVW, WHW/TL and LVW/TL in the SHR-EX group were decreased when compared with the SHR group (Table 1). The SBP, DBP, MAP, PP and heart rate were significantly elevated in the SHR group when compared with the WKY (Table 1). We observed that the SHR-EX group in the SBP and PP were lower about 10% and 25% more than the SHR group (Table 1). There were no significant differences in LVW/WHW among the three groups (Table 1).

**Table 1:** Cardiac characteristics of WKY, SHR, and SHR-EX groups.

**Parameters/Groups**	**WKY**	**SHR**	**SHR-EX**
Number	8	8	8
BW (g)	398 ± 14	407 ± 11	394 ± 11
WHW (g)	1.34 ± 0.11	1.61 ± 0.11^**^	1.46 ± 0.04^*#^
LVW (g)	1.01 ± 0.18	1.33 ± 0.07^**^	1.16 ± 0.07^*#^
WHW (g)/BW (kg)	3.35 ± 0.25	3.96 ± 0.28^**^	3.72 ± 0.19^*^
LVW (g)/BW (kg)	2.53 ± 0.46	3.28 ± 0.15^**^	2.94 ± 0.22
LVW/WHW	0.75 ± 0.12	0.83 ± 0.04	0.79 ± 0.04
WHW (g)/Tíbia (mm)	0.032 ± 0.003	0.039 ± 0.003^**^	0.035 ± 0.001^*#^
LVW (g)/Tíbia (mm)	0.024 ± 0.004	0.032 ± 0.002^**^	0.028 ± 0.002^*#^
SBP (mmHg)	113 ± 6	195 ± 6^**^	176 ± 4^**##^
DBP (mmHg)	78 ± 11	148 ± 8^**^	140 ± 14^**^
MAP (mmHg)	89 ± 10	163 ± 7^**^	152 ± 9^**^
PP (mmHg)	35 ± 8	47 ± 8^*^	35 ± 10^*#^
Heart rate	298 ± 28	356 ± 34^**^	345 ± 23^**^

Values are means ± standard deviation (SD). Three groups: Wistar-Kyoto rats (WKY), sedentary spontaneously hypertensive rats (SHR), and spontaneously hypertensive rats with exercise training (SHR-EX). Abbreviations: BW: body weight; WKW: whole heart weight; LVW: left ventricular weight; SBP: systolic blood pressure; DBP: diastolic blood pressure; MAP: mean arterial pressure; PP: pulse pressure. ^*^p < 0.05, ^**^p < 0.01, significant differences from the WKY group. ^#^*p* < 0.05, ^##^*p* < 0.01, significant differences between SHR-EX and SHR group.

### Cardiac histopathological changes

**Figure 1:**
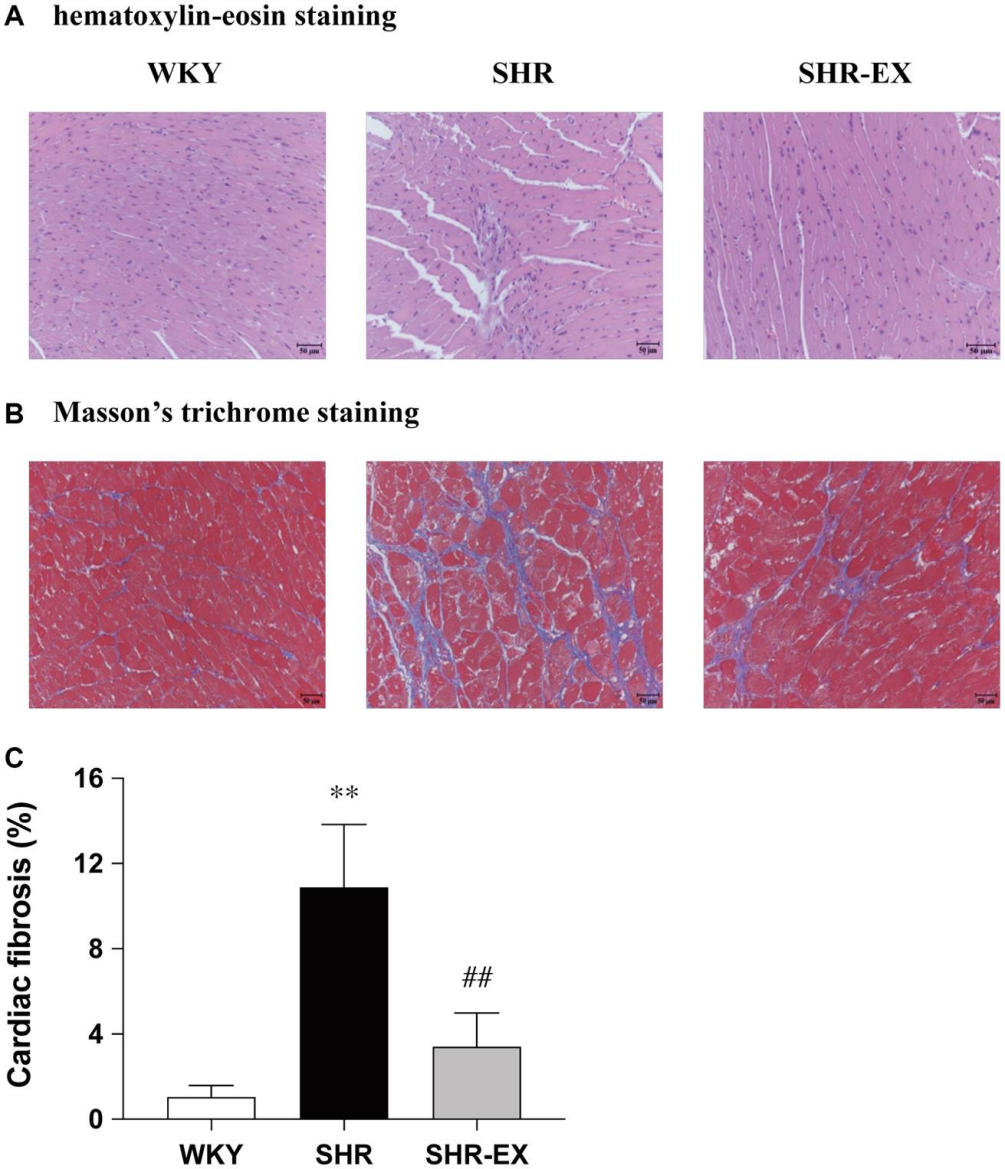
Representative histopathological analysis of cardiac tissue sections was performed with (**A**) hematoxylin-eosin staining (interstitial space: wide, arrows indicated) and (**B**) Masson’s trichrome staining (fibrosis: blue color, arrows indicated). The images of the myocardial architecture are magnified 200×; Bar scales = 50 μm. (**C**) The bar represents the percentage of blue area to the field area in Masson’s trichrome staining. Data are expressed as the mean values ± SD (*n* = 6 in each group). ^**^P < 0.01 vs. the WKY group. ^##^*P* < 0.01 the SHR group vs. SHR-EX group.

The paraffin-embedded sections of hearts from each group were stained by hematoxylin-eosin (H&E) staining and Masson’s trichrome staining to determine cardiac architecture and fibrosis from the WKY, SHR and SHR-EX groups. The abnormal myocardial architecture, enlarged myocardial interstitial space, and abnormal increase of interstitial fibers were significantly increased in the SHR group (Figure 1A–1C), which were reduced in the SHR-EX group when compared with the SHR group (Figure 1A–1C).

### Cardiac angiotensin II type I receptor and fibroblast growth factor 23

**Figure 2:**
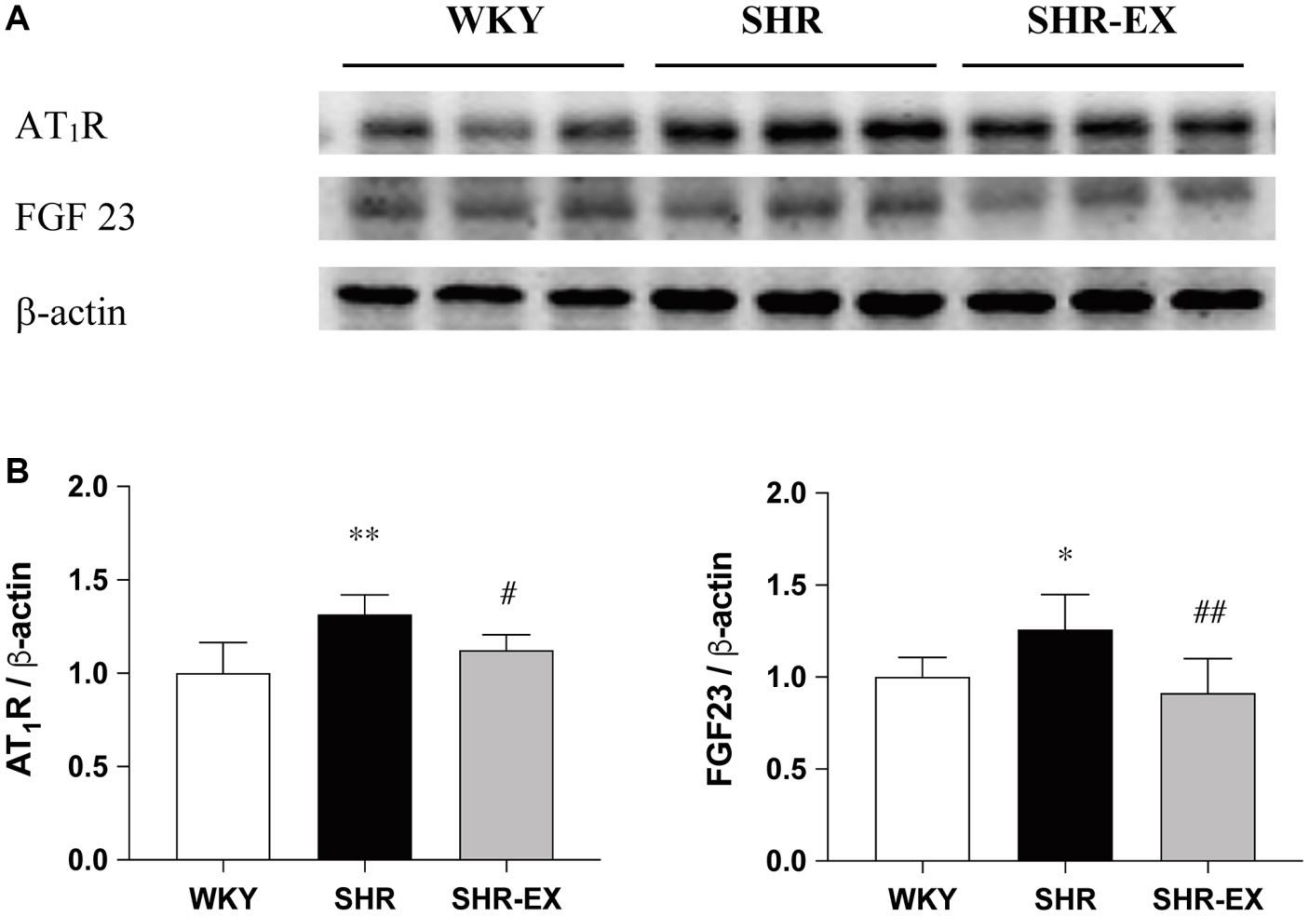
**Effects of exercise training on the AT_1_R and FGF23.** (**A**) The representative protein quantification of AT_1_R and FGF23 extracted from the left ventricles were measured by Western blotting analysis; (**B**) Bars represent the densitometric analysis of AT_1_R and FGF23. Data are expressed as the mean values ± SD (*n* = 8 in each group). ^*^p < 0.05, ^**^p < 0.01 vs. WKY group. ^#^*p* < 0.05, ^##^*p* < 0.01 the SHR group vs. SHR-EX group.

To investigate the components of the cardiac AT_1_R and FGF23 with Western Blot methods among WKY, SHR and SHR-EX groups. When compared with the WKY group, the AT_1_R and FGF23 protein levels were significantly increased in the SHR group, which were reduced in the SHR-EX group when compared with the SHR group (Figure 2).

### Cardiac fibrotic upstream pathways

**Figure 3:**
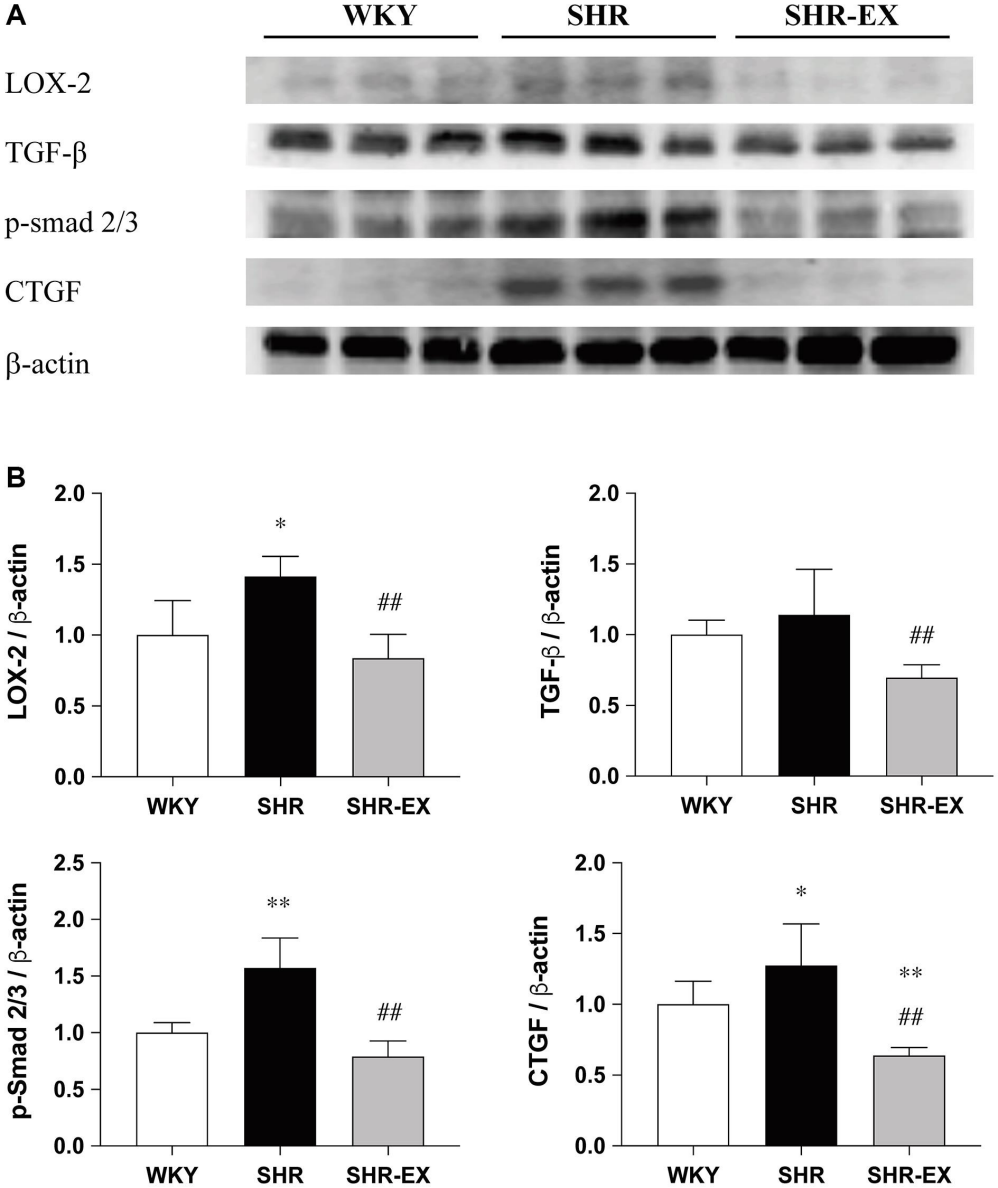
**Effects of exercise training on the cardiac fibrotic upstream pathway.** (**A**) The representative protein quantification of LOX-2, TGF-β, CTGF and p-Smad2/3 extracted from the left ventricles were measured by Western blotting analysis; (**B**) Bars represent the densitometric analysis of LOX-2, TGF-β, CTGF and p-Smad2/3. Data are expressed as the mean values ± SD (*n* = 8 in each group). ^*^p < 0.05, ^**^p < 0.01 vs. the WKY group. ^##^*p* < 0.01 the SHR group vs. SHR-EX group.

To investigate the cardiac fibrotic upstream pathways with Western Blot methods among the WKY, SHR and SHR-EX groups. The LOX-2, TGF-b, CTGF and p-Smad2/3 protein levels were significantly increased in the SHR group, when compared with the WKY control group, which were decreased in the SHR-EX group when compared with the SHR group (Figure 3).

### Cardiac fibrotic downstream pathways

**Figure 4:**
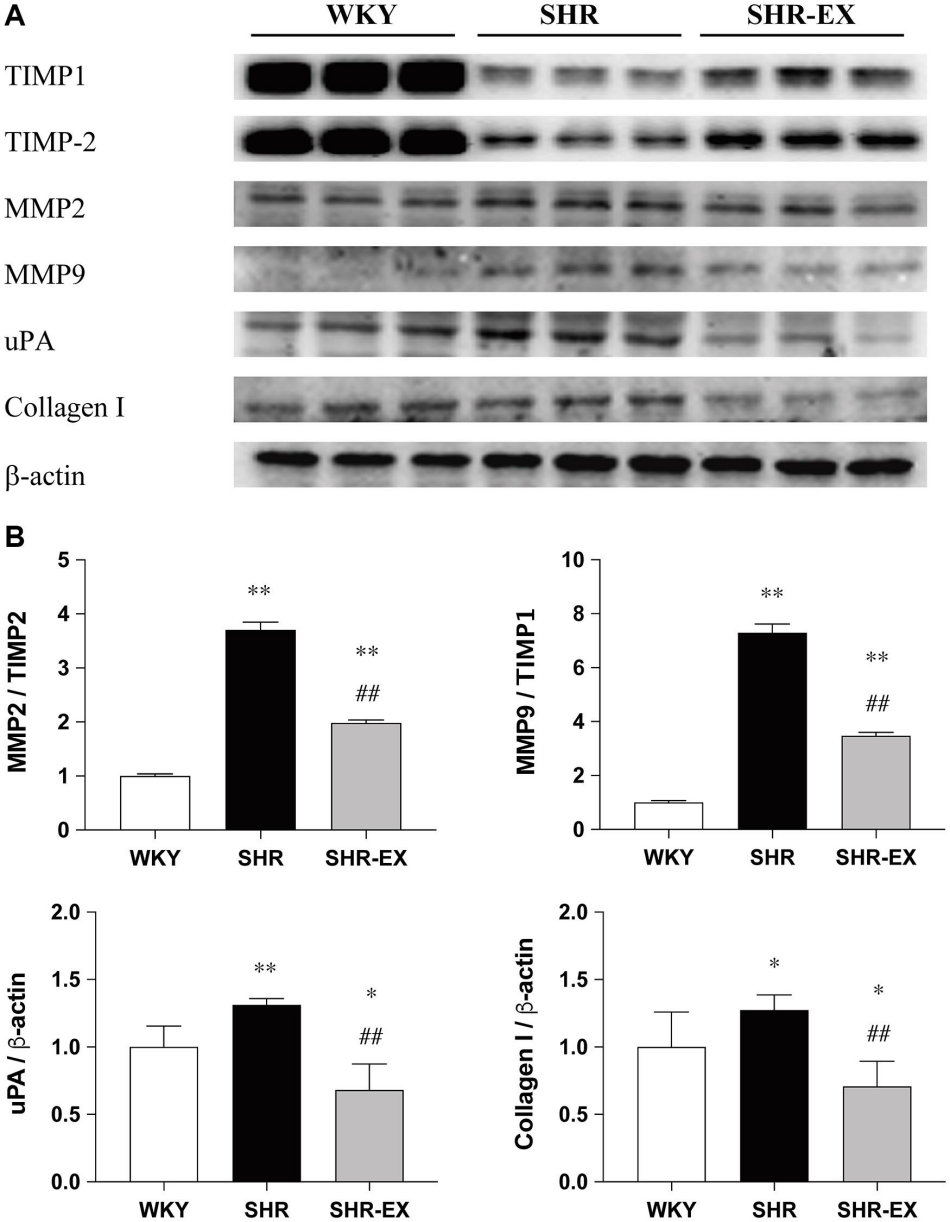
**The effects of exercise training on the cardiac fibrotic downstream pathway.** (**A**) The representative protein quantification of TIMP1, TIMP2, MMP2, MMP9, uPA and Collagen I extracted from the left ventricles were measured by Western blotting analysis; (**B**) Bars represent the densitometric analysis of MMP2/TIMP2, MMP9/TIMP1, uPA and Collagen I. Data are expressed as the mean values ± SD (*n* = 8 in each group). ^*^p < 0.05, ^**^p < 0.01 vs. the WKY group. ^##^*p* < 0.01 the SHR group vs. SHR-EX group.

To investigate the cardiac fibrotic downstream pathways with Western Blot methods among the WKY, SHR and SHR-EX groups. The MMP2/TIMP2, MMP9/TIMP1, uPA and Collagen I protein levels were significantly increased in the SHR group when compared with the WKY control group, which were decreased in the SHR-EX group when compared with the SHR group (Figure 4).

## DISCUSSION

**Figure 5:**
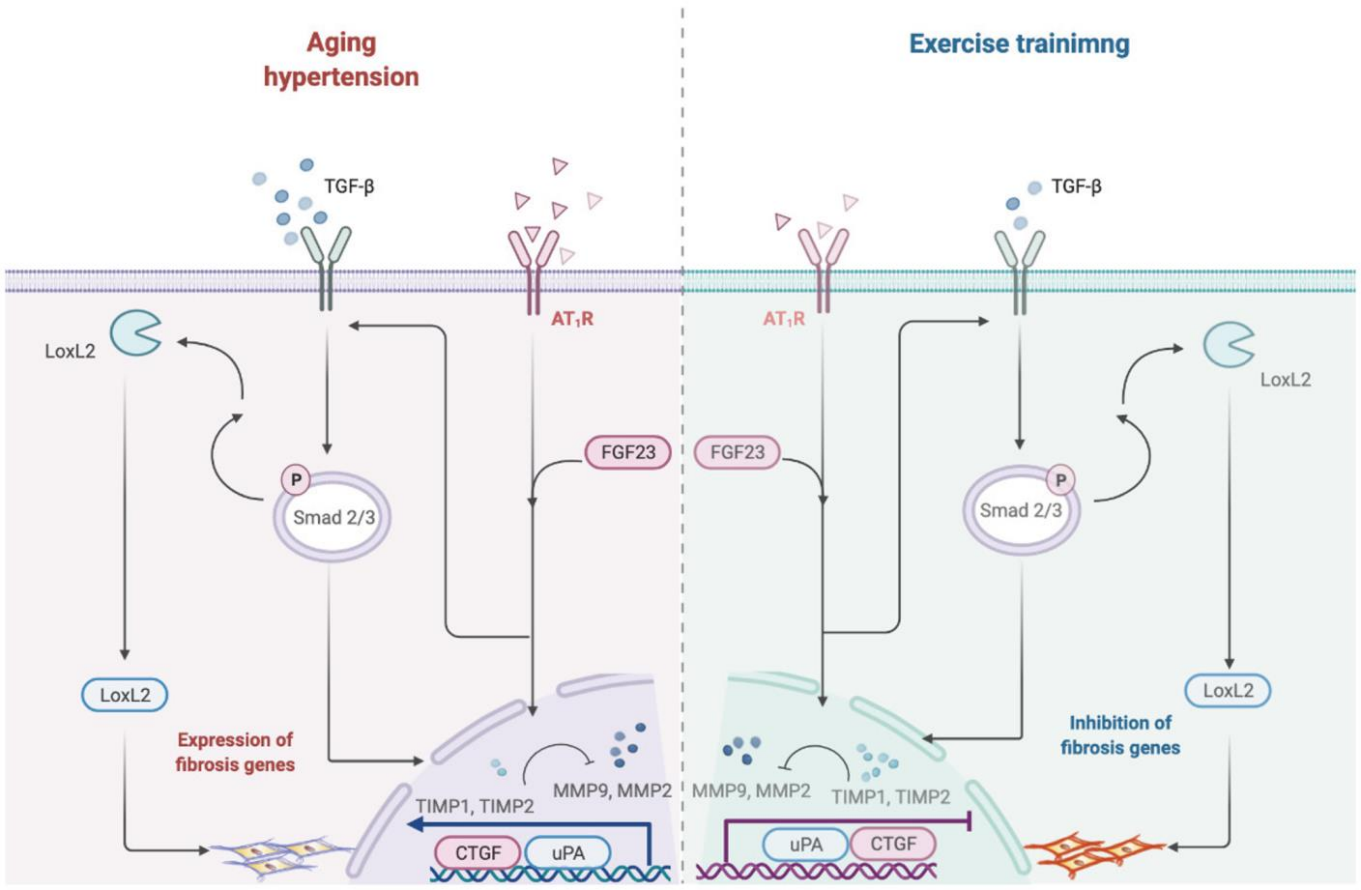
**Hypothesized diagram.** Schematic diagram from the present study showing that early aged hypertension activate the cardiac fibrotic upstream pathway (LOX-2, TGF-β, CTGF and p-Smad2/3), and activate the cardiac fibrotic downstream pathway (MMP2/TIMP2, MMP9/TIMP1, uPA and Collagen I) in the early aged hypertension. However, exercise training through the attenuated angiotensin II type I receptor and FGF23 and suppress cardiac LOXL2/ TGF-β-mediated fibrotic pathways on the early aged hypertensive heart.

The major findings of the present study are (1) Exercise training decreased early aged hypertensive heart caused high blood pressure, enlarged myocardial interstitial space, and abnormal increase of interstitial fibers. (2) Hypertension enhances the AT_1_R and FGF23 expression whereas the exercise training decreases cardiac AT_1_R and FGF23 expression. (3) Exercise training suppresses early aged hypertensive heart-induced fibrosis pathways such as AT_1_R, FGF23, LOX-2, TGF-β, CTGF, p-Smad 2/3, MMP-2/TIMP-2, MMP-9/TIMP-1, uPA and collagen I. From the current study did support our expected hypothesis that exercise training might attenuate myocardial fibrotic pathways through decreasing AT_1_R and FGF23 in early aged hypertensive rats. To sum up, cardiac fibrosis pathways could be less activated by exercise training, which is associated with and suppresses AT_1_R and FGF23 (Figure 5).

The SHR has many similarities with human hypertension, and large number of other pathophysiological phenotypes including left ventricular hypertrophy, insulin resistance and dyslipidemia [26]. Human and animal experiments have shown that myocardial interstitial fibrosis is an important pathologic mechanism, which is part of cardiac remodeling that leads to cardiac failure and sudden death [27]. Interstitial fibrosis plays an essential role in the development and progression of heart failure, which is affected by abnormal left ventricular relaxation and filling, ventricular stiffness [28]. In the present study, we observed that a considerably enlarged interstitial space was observed along with the deposition of bundles of collagen fibrils. This implies that, the presence of hypertension accelerates aging-related alterations of heart and arterial structure and function, and these two conditions substantially overlap in etiology and underlying mechanisms.

Several studies have shown that left ventricular hypertrophy and myocardial fibrosis linked to increase in AT_1_R in chronic angiotensin II-induced hypertension. In the heart, angiotensin II paracrine and autocrine action modulates myocardial growth via the AT_1_R, reflecting the stimulated generation of fibrosis-related pathway and/or the enhanced AT_1_R, the subsequent promotion of myocyte growth and myocardial fibrosis in the development of cardiac hypertrophy and heart failure (24). Moreover, FGF-23 may participate in hemodynamic and myocardial responses. One study reported that elevated FGF-23 exhibited increased blood pressure and left ventricular hypertrophy in angiotensin II treated animals [7]. However, a previous study indicated that endurance training-induced angiotensin II expression was increased and the AT_1_R expression was decreased, which may be a protective mechanism to avoid cardiac pathological hypertrophy [29]. Exercise was reported to attenuate pathological left ventricular hypertrophy, and regulation of the heart load and energy metabolism inducing the angiotensin II feedback and AT_2_R [30]. Our previous study displayed that aerobic exercise training on treadmills suppresses AT_1_R on hypertensive ovariectomized rats’ hearts. In our current study, we observed that treadmill exercise training could also decrease protein levels of AT_1_R and FGF-23 for the early aged hypertension. This implies that, in the pre-middle-aged hypertensive subjects, exercise training might attenuate myocardial fibrosis which is possibly associated with decreasing AT_1_R and FGF-23 expression.

Yang et al. has indicated identifying molecular pathways responsible for LOXL2 regulation will provide an opportunity to integrate myocardial fibrosis with mechanisms of heart failure and to identify additional new targets for heart failure therapy [9]. In the present study, it was found that LOXL2 and TGF-β were upregulated, indicating the activation of the LOXL2 signaling pathway in early aged hypertensive hearts. However, 12 weeks of aerobic exercise could significantly reduce the expression of LOXL2 and the downstream molecules in the heart of early aged hypertensive rats and this effect is related to the inhibition of myocardial fibrosis. A previous study has indicated that the TGF-β signaling pathway have been implicated in the pathogenesis of cardiac remodeling and fibrosis caused by myocardial infarction and hypertension [11]. Our data showed that the levels of TGF-β and CTGF were increased in early aged hypertensive hearts. Moreover, exercise in the present study may decrease the expression of TGF-β and CTGF in the heart of early aged hypertensive rats and contributed to reduce the degree of myocardial fibrosis in early aged hypertensive rats. This implies that, the LOXL2 could interact with TGF-β signaling pathway, which triggers formation of myofibroblasts with stimulated collagen deposition and crosslinking in the hypertrophic regions of early aged hypertensive hearts. However, exercise could decrease collagen deposition through LOXL2 and TGF-β -related fibrotic pathways.

The MMP/TIMP ratio is regarded as an independent predictor of cardiovascular disease severity [17]. Chiao et al. reported that MMP-9 involvement in cardiac ageing correlate with many different pathogeneses of left ventricle remodeling including increased left ventricle collagen deposition and a decline in diastolic function [31]. Ducharme et al. reported that the genetic deletion of MMP-9 attenuated collagen accumulation and dilation of the left ventricle post MI [14]. The current research observed that early aged hypertensive induction caused a significant increase in cardiac levels of MMP-9/TIMP-1 and MMP-2/TIMP-2 when compared to the age-match normotensive rats. Exercise training has been shown to provide anti-fibrotic effects as inferred from downregulated MMP-9/TIMP-1 and MMP-2/TIMP-2 by enhancing protective systems especially in the heart tissue. These data imply that the MMPs/TIMPs system is affected by high blood pressure and leads to widely dispersed apoptosis of the heart muscle cells and subsequently, fibrosis. Moreover, a TIMP/MMP pathway is a viable candidate pathway of exercise protection against aged hypertensive-related fibrosis.

This study has some limitations. A previous study indicated that the chronic loss of AT_2_R abolished cardiac hypertrophy and cardiac fibrosis [32]. Moreover, exercise could attenuate pathological myocardial hypertrophy, and regulation of the cardiac load and energy metabolism by the stimulation of the angiotensin II feedback and AT_2_R [30]. Further studies are required to evaluate these points. On the other hand, the face that exercise training has multiple beneficial effects on hearts cannot be isolated to one specific factor, such as oxidative stress [33], adrenergic system [34], or renin–angiotensin–aldosterone system [30].

In conclusion, these findings suggest that exercise training suppresses early aged hypertensive heart-induced LOX-2/TGF-β-mediated fibrotic pathways associated with decreasing AT1R and FGF23, which might provide a new therapeutic effect for exercise training to prevent adverse cardiac fibrosis and myocardial abnormalities in early aged hypertension.
